# Macrophages Promote Atherosclerosis Development by Inhibiting CD8T Cell Apoptosis

**DOI:** 10.1155/2024/1929766

**Published:** 2024-09-21

**Authors:** Xiaoming Xu, Yuteng Wu, Yifei Xu, Wei Mao, Yanyun Pan

**Affiliations:** ^1^ Department of Cardiology The First Affiliated Hospital of Zhejiang Chinese Medical University (Zhejiang Provincial Hospital of Chinese Medicine), Hangzhou 310058, China; ^2^ Department of Cardiology Affiliated Zhejiang Hospital Zhejiang University School of Medicine, Hangzhou 310058, China

## Abstract

**Background:**

Atherosclerosis is an inflammatory cardiovascular disease. However, whether the association of immune cells in plaques promotes the progression of this disease has not yet been completely elucidated.

**Materials and Methods:**

Thus, this study aimed to investigate the relationship between C1q+ macrophages and CD8T cells through scRNA-seq data reanalysis, quantitative real-time PCR, and flow cytometry. Chromatin immunoprecipitation-quantitative polymerase chain reaction, western blot, and antibody-blocking experiments were performed to investigate the role of macrophage–CD8T interaction in atherosclerosis. An atherosclerotic mouse model was developed to confirm our findings.

**Results:**

Mechanistically, Spi1 expression induced by granulocyte–macrophage colony-stimulating factor promoted C1q expression in the macrophages. Moreover, C1q+ macrophages suppressed CD8T cell apoptosis by upregulating Slc7a7 expression to enhance the L-arginine uptake of CD8T cells. CD8T-derived interferon-*γ* promoted macrophage activation to induce atherosclerosis. Blockade of the C1q–C1qbp axis attenuated atherosclerosis.

**Conclusion:**

In conclusion, macrophages interacting with CD8T promote atherosclerosis development via the C1q–C1qbp axis.

## 1. Introduction

Atherosclerosis is a systemic cardiovascular disease that is prevalent worldwide [1]. Chronic atherosclerosis causes ischemic cardiomyopathy, stroke, myocardial infarction, and peripheral arterial disease, which have high mortality rates [2]. At the initiation of atherosclerosis, endothelial cells in the normal artery are activated by proinflammatory cytokines or external stimuli and recruit blood-derived monocytes and lymphocytes into the endothelial layer by expressing VCAM-1 [3]. After these leukocytes migrate into the intima, macrophages that differentiate from blood-derived monocytes engulf lipids and are gradually converted to foam cells [4]. Other studies have demonstrated that foam cells can originate from smooth muscle cells [5]. Intima infiltrated by lymphocytes, principally CD8T cells, exerts cytotoxic effects and amplifies inflammation, which contributes to foam cell accumulation, endothelial damage, and subsequent plaque erosion [6]. Macrophages initiate T cell activation and differentiation by secreting pro-inflammatory factors, expressing MHC-peptide complexes during the host antimicrobial response [7], or suppressing the tumor antigen-specific CTL response by secreting metabolic byproducts and cytokines in the tumor microenvironment [8].

Complement C1q subcomponent subunits A/B/C (C1qa/b/c) are related to proenzymes C1r and C1s to yield C1, which is the first subcomponent of the C1 complex of the serum complement system [9]. C1q is expressed in different cell types, including monocytes, macrophages, dendritic cells, and cancer cells [10]. C1q+ macrophages play an immunosuppressive role in the tumor microenvironment by reprogramming fatty acid metabolism [11]. The C1q module of genes involved in the cell death pathway enhances the survival of macrophage-derived foam cells [12]. Our reanalysis of published scRNA-seq data also identified that C1q is highly expressed in plaque-derived macrophages. However, whether C1q+ macrophages affect other cell subsets expressing C1qR [13] or complement 1Q binding protein (C1qbp) [14] remains unclear. Moreover, the relationship between C1q+ macrophages and CD8T cells has been explored in some diseases but not in atherosclerosis.

Thus, this study aimed to investigate the relationship between C1q+ macrophages and CD8T cells by analyzing published scRNA-seq data and elucidate the mechanisms underlying their interactions by performing biological and molecular experiments. To the best of our knowledge, this is the first study to show that C1q+ macrophages prevent CD8T cell apoptosis by inducing Slc7a7-mediated L-arginine uptake, which exacerbates atherosclerosis development.

## 2. Materials and Methods

### 2.1. Cell Culture

Mouse peritoneal macrophages were induced by injecting thioglycolate medium (Millipore, Germany). Macrophages were cultured in RPMI 1640 complete medium supplemented with 10% fetal bovine serum (FBS; Gibco, Australia) at 37°C with 5% CO_2_ in a humidified incubator. Mouse primary spleen-derived CD8T cells were isolated using a mouse CD8T cell isolation kit (Miltenyi Biotec, Germany) and cultured in ALyS505N-0 medium supplemented with 10% FBS (Gibco) at 37°C with 5% CO_2_ in a humidified incubator. Macrophages were stimulated with 40 *μ*g/mL oxidized low-density lipoprotein (oxLDL; Yiyuan, China) or 14 nM DB2313 (HY-124629, MCE). After being stimulated with mouse T-activator anti-CD3/28 Dynabeads (Gibco), CD8T cells were used for subsequent experiments. The mice used above were anesthetized using sodium pentobarbital (50 mg/kg, i.p.; Canada), and euthanized using CO_2_ (100%) ventilation for 10 min. Then, mice were recognized as dead. Total 30 mice were used for macrophage isolation, and total 20 mice were used for CD8T cells isolation. No mice died during pre-experiment feeding stage. The duration of experiments ranges from 1 to 2 hr. Coculture experiments were performed as follows: macrophages were activated by treatment with interferon (IFN)-*γ* (10 ng/mL; #315-05, Peprotech) for 12 hr and then added into RPMI 1640 complete medium containing CD8T cells at a 1 : 1 ratio. For antibody blocking experiments, *α*-C1qbp (10 *μ*g/mL; ab24733, Abcam, USA), *α*-IFN-*γ* (#BE0055; Bioxcell), or *α*-IFNGR1 (#BE0287; Bioxcell) was added at the same time. After being cocultured for 24 hr, CD8T cells were collected for subsequent detection.

### 2.2. Western Blot

Macrophages were harvested after treatment and lysed on ice for 30 min using cell lysis buffer (CST, USA). The lysates were mixed with 5 × loading buffer (Beyotime, China), boiled for 10 min, run on a 4%–20% gradient SDS-PAGE gel, transferred onto polyvinylidene fluoride membranes (Millipore, USA), and then incubated overnight at 4°C with the following primary antibodies: anti-Stat1 (1 : 1,000; CST, #14994, USA), anti-pStat1 (Tyr701) (1 : 1,000; CST, #9167, USA), and anti-GAPDH (1 : 2,000; Abcam, ab181602). The membranes were washed three times with TBST for 15 min and then incubated with secondary antibodies (1 : 2,000, CST, USA) for 1 hr at room temperature. Finally, signals were detected using an enhanced chemiluminescence kit (Pierce, USA).

### 2.3. Quantitative Polymerase Chain Reaction (qPCR)

After being cocultured with CD8T cells, macrophages were harvested. Total RNA was extracted from the macrophages using Trizol reagent (Invitrogen, USA). RNA was reverse transcribed into cDNA using a PrimeScript RT Master Mix kit (Takara, Japan). cDNA of each sample was subjected to qRT‒PCR reactions consisting of 40 cycles of 95°C for 10 s, 68°C for 30 s, and 72°C for 30 s on a Q7 real-time PCR system (Applied Biosystems, USA) with SYBR Green Master Mix (Takara, Japan). The primers used were as follows: C1qa, 5′-TTCGGCAGAACCCAATGACG-3′ (forward), 5′-TGGTATGGACTCTCCTGGTTG-3′ (reverse); C1qb, 5′-ACGGCACGGAGGCTAAATTAT-3′ (forward), 5′-TCTTCGTGTCCAATCTCATCCT-3′ (reverse); C1qc, 5′-GGACGGGCATGATGGACTC-3′ (forward), 5′-TTCTGTTTGTATCGGCCCTCC-3′ (reverse); IL6, 5′-CTGCAAGAGACTTCCATCCAG-′ (forward), 5′-AGTGGTATAGACAGGTCTGTTGG-3′ (reverse); tumor necrosis factor (TNF), 5′-CAGGCGGTGCCTATGTCTC-3′ (forward), 5′-CGATCACCCCGAAGTTCAGTAG-3′ (reverse); and GAPDH, 5′-AGGTCGGTGTGAACGGATTTG-3′ (forward), 5′-GGGGTCGTTGATGGCAACA-3′ (reverse).

### 2.4. RNA Sequencing (RNA-Seq)

Macrophages or CD8T cells were harvested and lysed using Trizol reagent (Invitrogen). Total RNA from the macrophages or CD8T cells was extracted using Trizol reagent (Invitrogen). RNA-seq was performed using HiSeq 3000 (Illumina, USA) at Novogene (China). LifeScope v2.5.1 was used to align the reads to the genome, generate raw counts, and calculate the raw counts and reads per kilobase per million (RPKM) values. Differentially expressed genes (DEGs) were analyzed using the R package DESeq2 (version 1.12.3, https://rdocumentation.org/packages/DESeq2/versions/1.12.3), and enriched pathway analysis was performed using the R package clusterProfiler (version 3.0.4, https://rdocumentation.org/packages/clusterProfiler/versions/3.0.4).

### 2.5. Chromatin Immunoprecipitation (ChIP)-qPCR

After being cocultured with macrophages, CD8T cells were harvested and lysed using ChIP lysis buffer for 5 min on ice. ChIP was performed using a ChIP assay kit (Beyotime). After being sonicated to fragment chromatin, the lysates (5% was removed as input DNA) were incubated with 2 *μ*g of anti-RNA-poly II antibody (Abcam, ab193468), anti-H3K27ac antibody (Abcam, ab4729), or IgG (Millipore, USA) for 12 hr at 4°C with rotation and then with protein A + G agarose. The supernatant was washed three times successively with high-salt immune complex wash buffer, low-salt immune complex wash buffer, and LiCl immune complex wash buffer to extract the immunoprecipitated DNA–protein complex. qPCR detection was performed as described above. The primers used were as follows: Region-1, 5′-CTGAGTGGCTTTATTCTACTTAGTGACTGGG-3′ (forward); 5′-GAGTTTGCATCCTAGAGGTGTCATGGGC-3′ (reverse); Region-2 : 5′-GGCAAGTCTGTGTGGAGGAGTCTGC-3′ (forward), 5′-GCAGTGCTGAGCCTGCCAGAAAAGC-3′ (reverse). Region enrichment was calculated as follows: 2^-(Ct (each region from each sample) − Ct (each region from each input))^ × 100.

### 2.6. Flow Cytometry

After being cocultured with macrophages, CD8T cells were washed with ice-cold phosphate-buffered saline, stained with surface antigen-antibodies for 30 min at 4°C, and then subjected to flow cytometry using BD FACS Canto II. The following antibodies were used: v450-CD45 (75-0451-U100, Tonbo, USA), BV510-CD3e (100233, BioLegend, USA), Percp5.5-CD8a (65-0081-U100, Tonbo), PE-cy7-GZMB (25-8898-82, Invitrogen), APC-CD127 (60-1271-U100, Tonbo), FITC-KLRG1 (35-5893-U100, Tonbo), FITC-IFN*γ* (35-7311-U025, Tonbo), and APC-CD11b (20-0112-U025, Tonbo). Apoptosis was detected using a FITC Annexin V Apoptosis Detection Kit I (556547; BD Biosciences) in accordance with the manufacturer's instructions.

### 2.7. scRNA-Seq Analysis

Published mouse scRNA-seq data (GSE205930) and human scRNA-seq data (GSE179159) were integrated using a harmony algorithm [15]. After the Seurat object was created, scRNA-seq analysis was performed following the standard preprocessing workflow, including quality control, normalization, scaling, performing linear dimensional reduction, clustering the cells, running nonlinear dimensional reduction (Uniform Manifold Approximation and Projection, UMAP), finding differentially expressed features, and assigning cell type identity (https://satijalab.org/seurat/articles/pbmc3k_tutorial). Gene colocalization analysis was performed using the FeaturePlot function from the Seurat package with the blend parameter set as TRUE. Cell–cell communication was performed using CellChat [16] based on quantitative characterization and comparison of the inferred cell–cell communication networks using a systems approach combining social network analysis, manifold learning approaches, and pattern recognition. Transcription factor (TF) activities were evaluated using DoRothEA [17] based on the enrichment of a TF and its transcriptional targets in different cell subsets. Confidence was assigned to each TF-target interaction using a gene regulatory network.

### 2.8. Animal Experiments

*LDLR*−/− male mice on C57BL/6 background were purchased from JiCui Co. (Nanjing, China). Animal experiments were performed in accordance with the Ethics Committee of Zhejiang Chinese Medical University (ethical approval number: SYXK2021-0012). The mice (6 weeks old) were fed a high-fat diet for 12 weeks to induce atherosclerosis. Each group consisted of at least six mice. Total 20 mice were used in the study. Anti-C1qbp antibodies (100 *μ*g each time) or DB2313 (17 mg/kg each time) were intravenously injected two times per week. The aorta was isolated as follows. The mice used above were anesthetized using sodium pentobarbital (50 mg/kg, i.p.; Canada), and euthanized using CO_2_ (100%) ventilation for 10 min. Then, mice were recognized as dead. No mice died during pre-experiment feeding stage. The duration of experiments ranges from 2 to 4 hr. After abdominal cavities were exposed. The right atrium was opened, and then 5 mL of saline was injected into the left ventricle to wash the blood. Then, 4% polyformaldehyde was injected for 30 min to fix the tissue morphology. The aorta and perivascular adipose tissue were removed, and then the pulmonary arteriovenous malformation was excised. The artery and heart were removed and then placed in 4% paraformaldehyde at room temperature overnight. Finally, whole aortas were opened longitudinally and stained for lipids using Oil Red O and hematoxylin–eosin (Jiancheng Bio., China).

### 2.9. Statistical Analysis

All experiments were performed in triplicates. Data were statistically analyzed using R-4.3.2. Significant differences between two groups were determined using unpaired two-tailed Student's *t*-test. Significant differences among multiply groups were determined using ANOVA (parametric). Statistical significance was considered at *p* < 0.05, as indicated by an asterisk ( ^*∗*^).

## 3. Results

### 3.1. Association between Macrophages and CD8T Cells Was Strengthened in Atherosclerotic Mice

**Figure 1:**
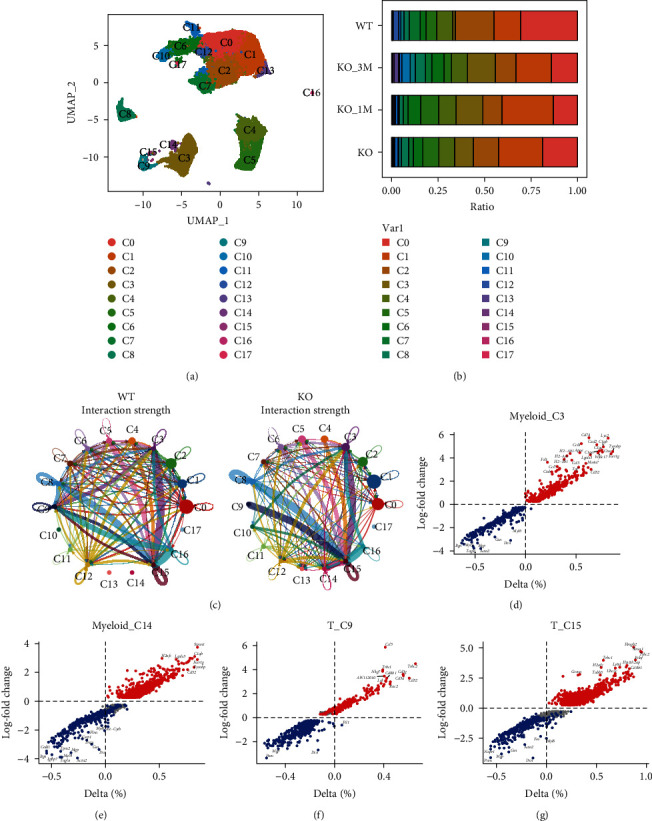
Macrophages interact with CD8T cells in atherosclerotic plaques: (a) UMAP where cells are clustered using functions of the Seurat package; (b) differential analysis of cluster ratio across four groups; (c) cluster interaction analysis between the WT and KO groups using CellChat; and (d–g) differentially expressed genes in macrophages (cluster 3, (d); cluster 14, (e)) or CD8T cells (cluster 9, (f); cluster 15, (g)).

Published scRNA-seq (GSE205930) data from wild-type (WT) and *LDLR*−/− mice-derived plaques were reanalyzed through UMAP dimension reduction. A total of 18 clusters were obtained (Figure 1(a)), among which C3 and C14 were recognized as myeloid cell subsets (marked by *Lyz2* and *Itgam*) and C9 and C15 were recognized as T cell subsets (marked by *Cd3e*, *Cd3g*, and *Cd3d*) (Figure S1(a,b)). As shown in Figure 1(b), the immune cell subsets (C3, C9, C14, and C15) were induced following atherosclerosis progression. Moreover, the interactions among immune cells were strengthened in the atherosclerotic mice (Figure 1(c)). DEG analysis indicated that the myeloid subsets (C3 and C14) highly expressed C1q family genes (*C1qa*, *C1qb*, and *C1qc*, Figures 1(d) and 1(e)), and the T cell subsets (C9 and C15) highly expressed *Lck*, *Nkg7*, *Cd8a1*, and *Gzma*, suggesting that these T cells mainly activated CD8T (Figures 1(f) and 1(g)). These data showed that communications between myeloid and CD8T cells were induced in the atherosclerotic mice.

### 3.2. Transcription Factor Spi1 Promoted C1q+ Macrophage Differentiation

**Figure 2:**
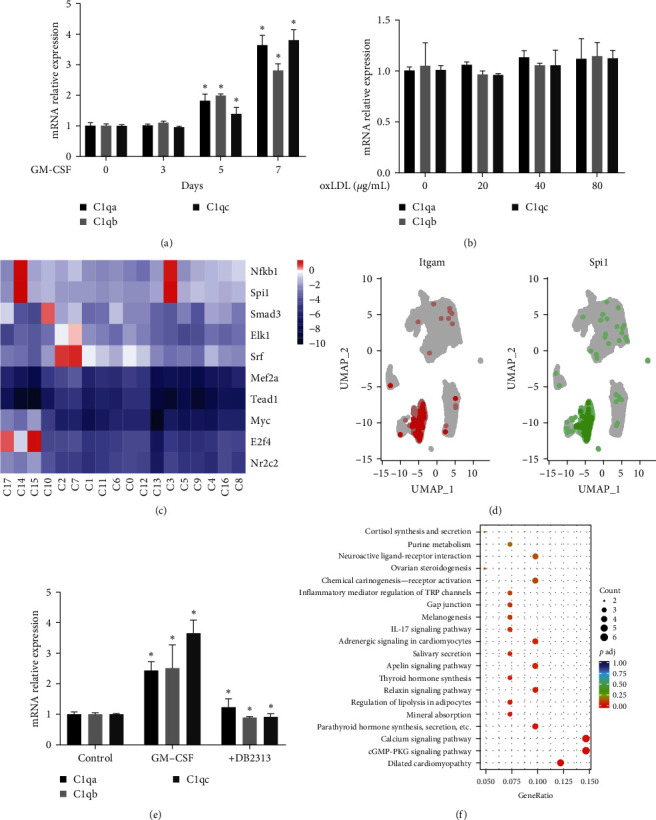
Spi1 induces C1q expression in macrophages. (a) Monocytes were treated with GM-CSF at the indicated time points. C1q was detected using qPCR. (b) Macrophages were treated with oxLDL at the indicated concentrations. C1q was detected using qPCR. (c) Heatmap showing differential analysis of the enriched transcription factors in clusters using the DoRothEA package. (d) UMAP plots showing the coexpression of Itgam and Spi1 in macrophage subsets. (e) Monocytes were treated with GM-CSF with or without DB2313 for 7 days. C1q was detected using qPCR. (f) Bubble plot showing KEGG enrichment of differential pathways in DB2313-treated macrophages compared with the control group.  ^*∗*^*p* < 0.05.

To investigate how C1q+ macrophages were induced, monocytes were first stimulated with granulocyte–macrophage colony-stimulating factor (GM-CSF). Consistent with our hypothesis, incubation with GM-CSF induced the expression of C1q genes (Figure 2(a)). However, oxLDL treatment did not induce the expression of C1q genes (Figure 2(b), Figure S1(c)), indicating that C1q+ macrophages were mainly derived from monocyte differentiation in the plaques. DoRothEA [18] results showed that the activities of TFs Spi1 and Nfkb1 were specifically high in C3 and C14 (Figure 2(c)). Gene colocalization analysis also revealed that Spi1 was highly expressed in the macrophages (Figure 2(d)). Treatment with Spi1 inhibitor DB2313 reversed the GM-CSF-induced expression of C1q genes, confirming that Spi1 mediated the transcription of C1q genes (Figure 2(e)). Meanwhile, RNA-seq results showed that treatment with DB2313 regulated cGMP-PKG (cyclic guanosine monophosphate-protein kinase G) signaling and calcium signaling (Figure 2(f)), indicating that Spi1 was essential for the basal activation of macrophages. These results indicated that Spi1 specifically induced the expression of C1q genes in the macrophages during differentiation.

### 3.3. C1q+ Macrophages Inhibited CD8T Apoptosis by Promoting L-Arginine Uptake

**Figure 3:**
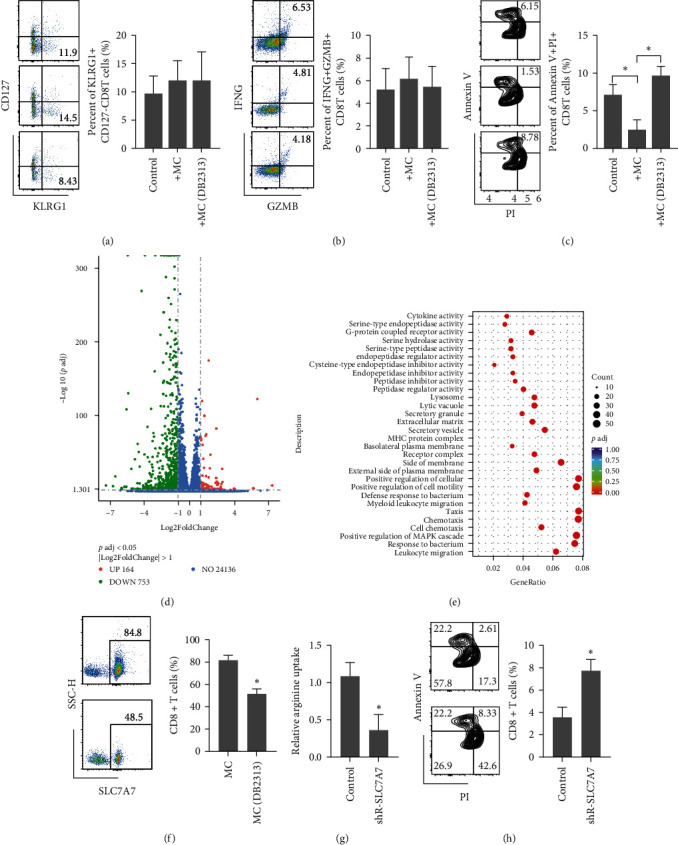
C1q+ macrophages prevent CD8T activation-induced apoptosis. (a–c) CD8T cells were activated using anti-CD3/CD28 beads before being cocultured with C1q+ macrophages treated with or without DB2313. Memory/effector differentiation (a), cytotoxicity (b), and apoptosis (c) were detected via flow cytometry. (d) Volcano plot showing the differentially expressed genes in CD8T cells cocultured with C1q+ macrophages treated with or without DB2313. (e) Bubble plot showing KEGG enrichment of differential pathways in CD8T cells cocultured with C1q+ macrophages treated with or without DB2313. (f) CD8T cells were activated using anti-CD3/CD28 beads before being cocultured with C1q+ macrophages treated with or without DB2313. SLC7A7 expression was detected using flow cytometry. (g) Relative ^3^H-arginine uptake into control and shR-SLC7A7-infected CD8T cells. (h) CD8T cells were activated using anti-CD3/CD28 beads with or without shR-SLC7A7 lentivirus infection. Apoptosis was detected via flow cytometry.  ^*∗*^*p* < 0.05.

These results identified C1q+ macrophages interacting with T cells in the plaques. We investigated whether this association affects the functions of T cells. Coculture of C1q+ macrophages (pretreated with DB2313 or not) and activated CD8T cells revealed that the macrophages did not affect memory differentiation (Figure 3(a)) or CD8T cytotoxicity (Figure 3(b)) of CD8T. However, DB2313-treated macrophages induced the apoptosis of CD8T cells (Figure 3(c)), suggesting that the C1q+ macrophages suppressed the apoptosis of CD8T cells. To explore the transcriptional changes in CD8T cells during coculture with C1q+ macrophages, we performed RNA-seq analysis. As shown in Figure 3(d), 164 genes were upregulated, while 753 genes were downregulated. Moreover, Kyoto Encyclopedia of Genes and Genomes pathway analysis showed that chemotaxis, amino acids, G protein-coupled receptors, and receptor complex pathways were enriched, indicating that the C1q+ macrophages affected the metabolism of CD8T cells (Figure 3(e)). Among these DEGs, the expression of Slc7a7 (solute carrier family 7 member 7, transport of L-arginine) was significantly reduced in the DB2313 group (Figure 3(f)). As expected, L-arginine uptake decreased (Figure 3(g)) and apoptosis increased in the CD8T cells (Figure 3(h)) upon Slc7a7 depletion, while DB2313 treatment alone had no significant effect on L-arginine uptake and apoptosis directly (Figure S1(d,e)). Taken together, these results indicate that the C1q+ macrophages suppressed CD8T cell apoptosis by inducing L-arginine uptake mediated by SLC7A7.

### 3.4. C1q–C1qbp Axis Activated SLC7A7 Transcription and Sustained Mitochondrial Homeostasis

**Figure 4:**
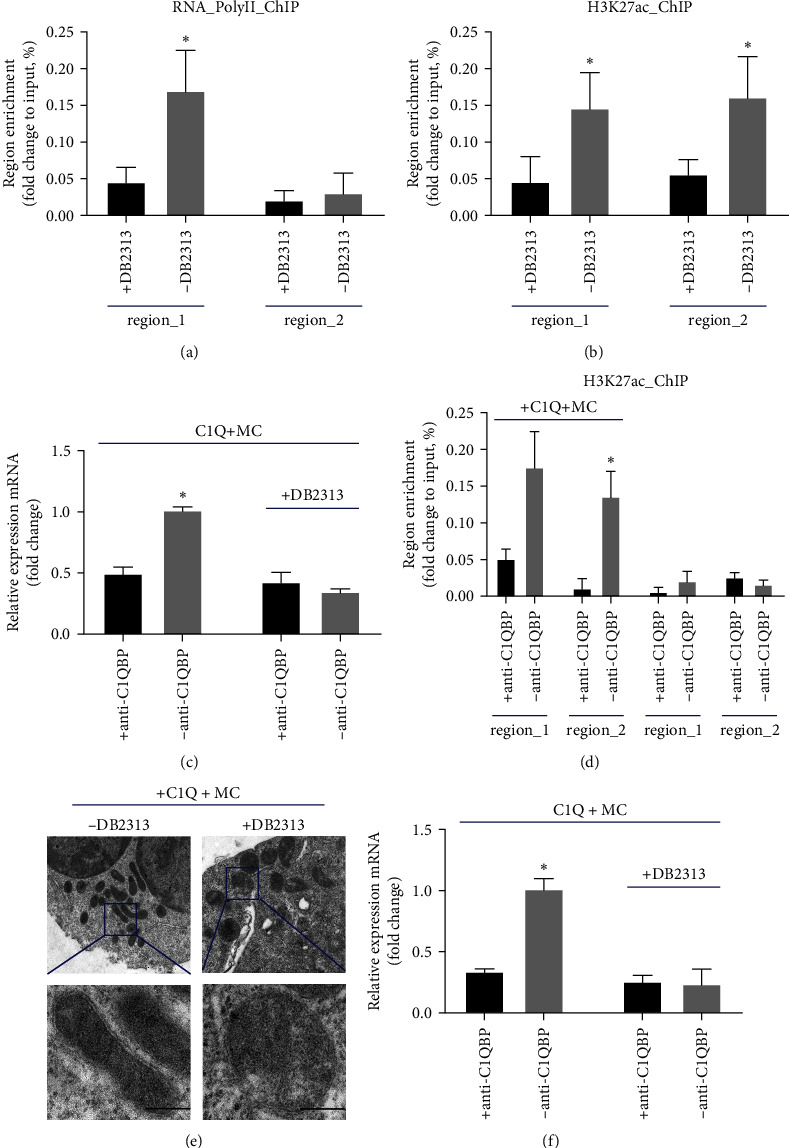
C1q+ macrophages induce SLC7A7 expression in CD8T cells dependent on H3K27ac modifications. (a, b) CD8T cells were activated using anti-CD3/CD28 beads before being cocultured with C1q+ macrophages treated with or without DB2313. Pol II abundance (A) or H3K27ac modifications at different regions of the SLC7A7 promoter were detected using a chromatin immunoprecipitation (ChIP) assay. (c) CD8T cells were activated using anti-CD3/CD28 beads before being cocultured with C1q+ macrophages treated with or without anti-C1QBP. SLC7A7 expression was measured using qPCR. (d) CD8T cells were activated using anti-CD3/CD28 beads before being cocultured with C1q+ macrophages treated with or without anti-C1QBP. H3K27ac modifications at different regions of the SLC7A7 promoter were detected using a chromatin immunoprecipitation (ChIP) assay. (e) Representative electron micrographs of mitochondria in CD8T cells activated using anti-CD3/CD28 beads before being cocultured with C1q+ macrophages treated with or without DB2313. Scale bars, 200 nm. (f) CD8T cells were activated using anti-CD3/CD28 beads before being cocultured with C1q+ macrophages treated with or without anti-C1QBP. C1QBP expression was measured using qPCR.  ^*∗*^*p* < 0.05.

Previous data showed that C1qbp expressed in T cells could be bound by C1q [19]. We suspected that the C1q–C1qbp axis mediates *Slc7a7* expression in CD8T cells. Coculture and ChIP-qPCR results showed that the C1q+ macrophages induced RNA polymerase II binding and H3K27ac modification on the *Slc7a7* promoter (Figures 4(a) and 4(b)). Consistent with our hypothesis, the anti-C1qbp antibody canceled the promoting effect of the C1q+ macrophages on *Slc7a7* transcription (Figures 4(c) and 4(d)). Moreover, the C1q+ macrophages promoted mitochondrial cristae formation (Figure 4(e)) and C1qbp transcription (Figure 4(f)), suggesting that the C1q–C1qbp axis promoted *Slc7a7* transcription and *C1qbp* transcription positive feedback to sustain mitochondrial homeostasis [20]. In conclusion, the C1q–C1qbp axis mediated the promoting effect of macrophages on *Slc7a7* expression in the CD8T cells.

### 3.5. CD8T-Derived IFN-*γ* Induced Macrophage M1 Polarization

**Figure 5:**
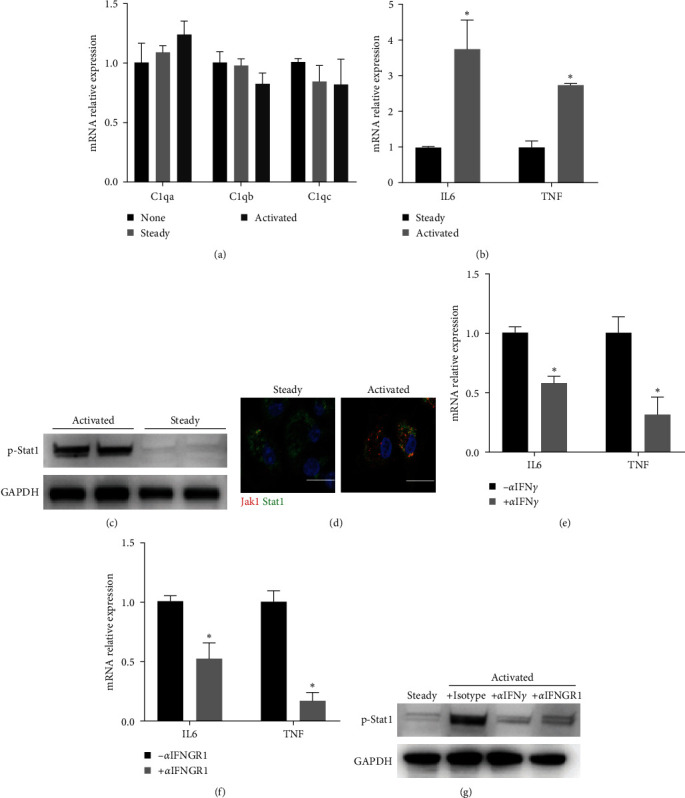
IFN-*γ* originated from activated CD8T promotes the secretion of JAK-STAT1 signaling-dependent proinflammatory factors in macrophages. (a) Macrophages were cocultured with activated or steady CD8T. C1qa, C1qb, and C1qc were detected using qPCR. (b) Macrophages were cocultured with activated or steady CD8T. IL6 and TNF levels were measured using qPCR. (c) Macrophages were cocultured with activated or steady CD8T. Phosphorated Stat1 in macrophages was detected using western blot, with GAPDH as an endogenous control. (d) Macrophages were cocultured with activated or steady CD8T. The cells were fixed, immunoblotted with anti-Jak1 (red) and anti-Stat1 antibodies (green) and DAPI (blue), and visualized using confocal microscopy. Scale bars, 20 *μ*m. (e, f) Macrophages were cocultured with activated or steady CD8T with or without anti-IFN-*γ* or anti-IFNGR1. IL6 and TNF levels were measured using qPCR. (g) Macrophages were cocultured with activated or steady CD8T with or without anti-IFN-*γ* or anti-IFNGR1. Phosphorated Stat1 in macrophages was detected using western blot, with GAPDH as an endogenous control.  ^*∗*^*p* < 0.05.

To investigate whether coculture with CD8T induces C1q+ macrophages, we performed coculture experiments with and without CD8T. C1q genes were not induced by the CD8T coculture (Figure 5(a)). Interestingly, pro-inflammatory factors interleukin-6 (IL-6) and TNF were clearly induced (Figure 5(b)). Considering that IFN-*γ* can be secreted by activated CD8T, we doubted whether IFN-*γ* signaling mediated this effect. As expected, coculture with activated CD8T cells promoted Stat1 phosphorylation (Figure 5(c)) and colocalization between Jak1 and Stat1 (Figure 5(d)). Blockade of IFN-*γ* signaling using *α*-IFN*γ* (Figure 5(e)) or *α*-IFNGR1 (Figure 5(f)) reversed the activated CD8T-induced expression of IL-6 and TNF, and Stat1 phosphorylation (Figure 5(g)). In conclusion, CD8T-derived IFN-*γ* induces macrophage M1 polarization during interaction between these two immune cells.

### 3.6. Blockade of the C1q–C1qbp Axis Attenuated Atherosclerosis Development

**Figure 6:**
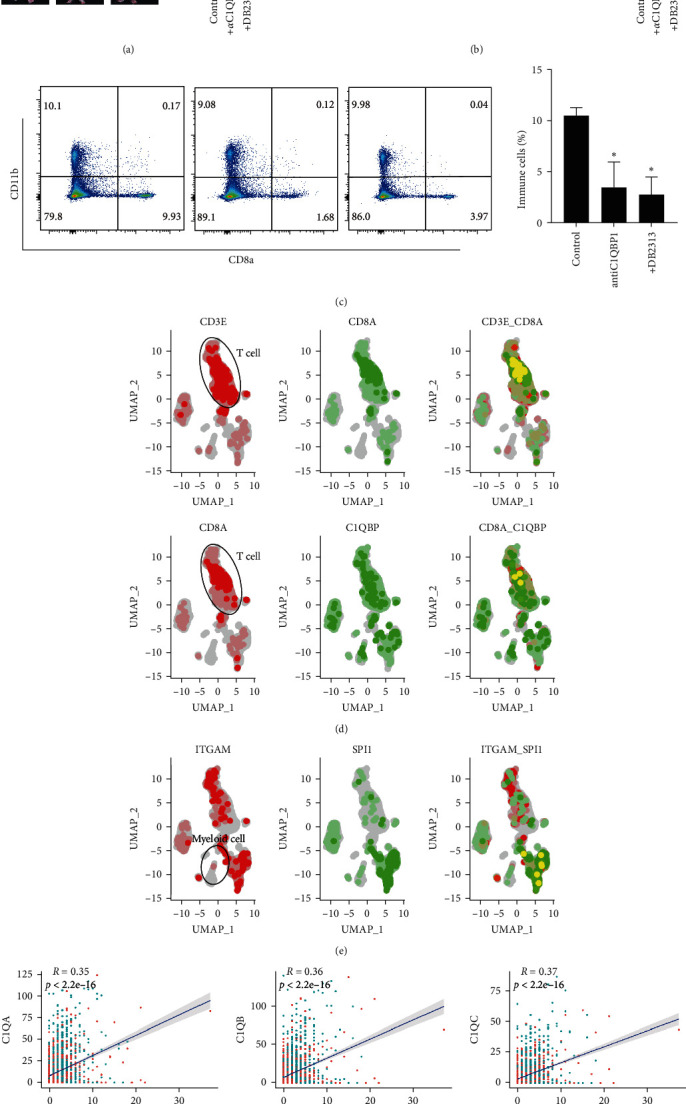
Blockade of the C1q–C1qbp axis impairs atherosclerosis development in mice: (a, b) representative images of the aorta en face lesion (A) or the aortic root lesion area (B) stained with oil red O across three groups. Scale bars, 0.5 cm (A). Scale bars, 1 mm (B). (c) Plaque-infiltrated CD8T in atherosclerotic mice with or without anti-IFN-*γ* or DB2313 injection was detected using flow cytometry. (d) UMAP plots showing the coexpression of CD8A, CD3E, and C1QBP in CD8T subsets from human scRNA-seq data. (e) UMAP plots showing the coexpression of ITGAM and SPI1 in macrophage subsets from human scRNA-seq data. (f) Scatter plot showing the correlation between SPI1 and C1QA, C1QB or C1QC in macrophage subsets from human scRNA-seq data.  ^*∗*^*p* < 0.05.

We explored the role of the C1q–C1qbp axis in atherosclerosis progression. Using *α*-C1qbp or DB2313 to specifically block the C1q–C1qbp axis, we found that the areas of plaques in the aorta decreased (Figure 6(a)) and that lipid accumulation around the aortic valve was reduced (Figure 6(b)). However, serum calcium and phosphorus contents did not change (Figure S1(f)) [21]. Furthermore, CD8T cell infiltration into the plaques was diminished (Figure 6(c)). Therefore, we aimed to elucidate the role of the C1q–C1qbp axis in human atherosclerotic plaques. We reanalyzed the published human plaque scRNA-seq dataset (GSE179159). As shown in Figures 6(d) and 6(e), C1QBP+ CD8T cells and SPI1+ macrophages were highly enriched in the human plaque samples. Importantly, SPI1 expression was positively correlated with C1QA (*R* = 0.35), C1QB (*R* = 0.36), and C1QC (*R* = 0.37) in the macrophage subsets (Figure 6(f)). Overall, the C1q–C1qbp axis exerted a positive effect on atherosclerosis.

## 4. Discussion

Macrophages promote atherosclerosis progression by producing foam cells through the uptake of low-density lipoproteins during different phases of atherosclerosis [22]. Classical complement C1q expression is enhanced in advanced atherosclerotic plaques and associated with plaque instability [23]. Serum C1q activity is higher in obstructive coronary artery disease and nonobstructive coronary artery disease than in the control group [24]. Additionally, C1q regulates macrophage polarization [25]. In the present study, we explored the role of C1q+ macrophages in preventing the apoptosis of CD8T cells for the first time. Through binding to the C1qbp receptor on the CD8T cells, the C1q+ macrophages promoted H3K27ac-mediated SLC7A7 transcription. The upregulated expression of SLC7A7 in the CD8T cells induced L-arginine uptake and inhibited CD8T cell apoptosis. These results help us to deeply understand the role of the crosstalk between macrophage and CD8T cells during atherosclerosis compared with existing data.

This study clarifies the role of the interaction between macrophages and CD8T cells in atherosclerosis, but it also has some limitations. First, C1q was induced by Spi1 following monocyte differentiation into macrophages under GM-CSF stimulation based on our experiments. However, whether C1q is also induced in dendritic cells, the strongest antigen-presenting cells that initially activate CD8T remains unclear. Second, intervention experiments were performed in an atherosclerotic mouse model using *α*-C1qbp or DB2313. However, the C1q–C1qbp axis is also extensively expressed in other immune cells; thus, our expectation of specifically blocking the C1q–C1qbp axis between macrophages and CD8T cells was discounted. Third, RNA-seq results of CD8T cells indicated changes in multiple biological activities, including peptidase inhibitor activity, chemotaxis, and leukocyte migration. Thus, C1q+ macrophages have other promoting effects on CD8T cells [26, 27].

Previous studies have shown that the elevation of L-arginine levels in CD8T promotes a metabolic shift from glycolysis to oxidative phosphorylation [14, 28]. Whether other L-arginine-dependent metabolic activities or molecular signaling pathways are also altered in CD8T cells warrants further investigation. Our data reanalysis confirmed the expression of C1qbp in myeloid cell subsets and other parenchymal cell subsets [29, 30]. However, whether the C1q–C1qbp axis between other cell subsets also promotes atherosclerosis remains to be clarified. Establishing a cell type-specific C1q knockout mouse model may contribute to answering these questions. Moreover, the present data were all based on transcriptional data. Further studies should characterize the C1q–C1qbp axis in human samples through immunofluorescence.

In conclusion, this study is the first to explore the relationship between C1q+ macrophages and CD8T cells in atherosclerotic plaques. Through a series of experiments, we demonstrated that C1q+ macrophages suppress the apoptosis of CD8T cells, which induce atherosclerosis.

## Data Availability

Raw RNA-Seq data were deposited in the NCBI with accession numbers GSE245183 and GSE245667. All the data from this study have been included in the manuscript.

## Supplementary Materials

Supplementary Materials Figure S1: identification of macrophages and T subsets.
