# Supplementary material for: Macrophages Promote Atherosclerosis Development by Inhibiting CD8T Cell Apoptosis
Source: Mediators Inflamm. 2024 Sep 21;2024:1929766. doi: 10.1155/2024/1929766 (PMC11438514; doi:10.1155/2024/1929766)
Supplement: Supplementary Materials — Figure S1: identification of macrophages and T subsets. [file 1929766.f1.docx]

**
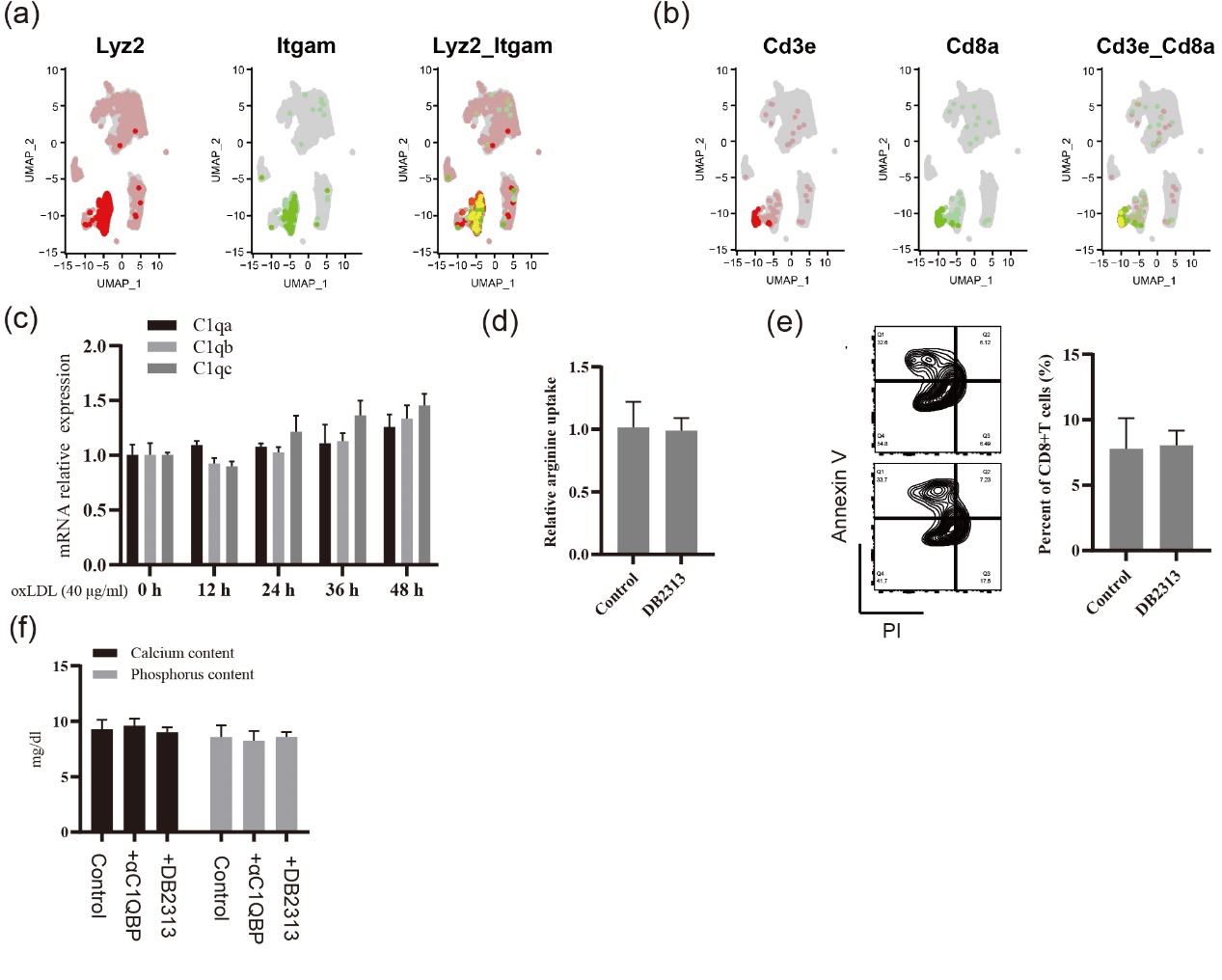
Figure S1: Identification of macrophages and T subsets.**

1. UMAP plots showing the co-expression of Lyz2 and Itgam in macrophage subsets.
2. UMAP plots showing the co-expression of Cd3e and Cd8a in T subsets.
3. Macrophages were treated with oxLDL at the indicated time points. C1q was detected using qPCR.
4. Relative ^3^H-arginine uptake into control and DB2313-treated CD8T cells.
5. CD8T cells were activated using anti-CD3/CD28 beads with or without DB2313 treatment. Apoptosis was detected via flow cytometry.
6. Calcium and phosphorus contents were detected in the serum of an atherosclerotic mouse model with the indicated treatments.
